# Clinical and Genetic Spectrum of *OPN1LW*/*OPN1MW*-Associated Disorders in a Chinese Cohort: Contribution of Long-Read Sequencing

**DOI:** 10.3390/ijms27219705

**Published:** 2026-10-29

**Authors:** Xing Wei, Yamei Li, Hui Li, Wuyi Li, Yunyu Zhou, Ruifang Sui

**Affiliations:** State Key Laboratory of Complex Severe and Rare Diseases, Department of Ophthalmology, Peking Union Medical College Hospital, Chinese Academy of Medical Sciences, Peking Union Medical College, No. 1, Shuai Fu Yuan, Beijing 100730, Chinawuyili651@outlook.com (W.L.);

**Keywords:** *OPN1LW*, *OPN1MW*, long-read sequencing, blue cone monochromacy, color-vision disorders

## Abstract

The highly homologous *OPN1LW*/*OPN1MW* gene cluster poses substantial challenges for molecular diagnosis. We retrospectively enrolled 12 male patients from 10 unrelated Chinese families with clinically suspected X-linked cone-opsin disorder, all of whom were confirmed to carry disease-associated variants or haplotypes in the *OPN1LW/OPN1MW* gene cluster. Clinical records, retinal imaging, electrophysiology, and available ocular biometry were analyzed together with short-read sequencing, Sanger validation, copy-number analysis, and Oxford Nanopore long-read sequencing for three cases with incomplete short-read resolution. Clinical subgroups included blue cone monochromacy (*n* = 5), X-linked cone dystrophy (*n* = 4), Bornholm eye disease (*n* = 2), and isolated color-vision deficiency (*n* = 1). Molecular results encompassed sequence variants, exon dosage alterations, a 51.8 kb deletion involving the locus control region, and exon 3 haplotypes. Long-read sequencing defined the deletion breakpoint in one case and the exon 3 haplotypes in two. In one boy with an X-linked cone dystrophy phenotype, the mean binocular axial length increased from 25.36 mm to 28.47 mm between 1.80 and 6.24 years of age. Our findings demonstrate the complementary utility of multimodal molecular assays and heterogeneous phenotypes of cone-opsin disorders, with sustained childhood axial elongation reported only as a single-case observation.

## 1. Introduction

Human trichromatic vision depends on short-, middle-, and long-wavelength-sensitive cone opsins. *OPN1SW* is located on chromosome 7, whereas *OPN1LW* and *OPN1MW* form a tandem gene cluster on Xq28, typically comprising one upstream *OPN1LW* and one or more *OPN1MW* copies. An upstream locus control region (LCR) regulates expression; gene order and structural integrity are therefore important for L/M-cone function [1,2,3,4].

High-sequence homology predisposes this locus to unequal recombination, gene conversion, exon exchange, copy-number variation, hybrid genes, and structural rearrangements. Disease-associated alterations range from single-nucleotide changes and small insertions or deletions to exon-level deletions, LCR disruption, and complex array rearrangements [2,5,6,7,8,9]. Associated phenotypes include red–green color vision deficiency, blue cone monochromacy (BCM), X-linked cone dystrophy (XLCOD), Bornholm eye disease (BED), cone or cone-rod dystrophy, and early-onset high myopia [5,7,10,11,12,13,14,15,16,17,18]. These overlapping phenotypes raise questions about how distinct molecular defects influence cone function and ocular development.

Short-read next-generation sequencing (NGS) can identify many sequence-level variants but may not resolve gene order, copy number, hybrid genes, or LCR involvement at this repetitive locus [8,9]. Long-read sequencing can span homologous regions and clarify disease-relevant array architecture [6,8,9]. We combined NGS, Sanger validation, segregation analysis, and Oxford Nanopore sequencing in selected cases to characterize 12 affected males from 10 families. We assessed the molecular findings alongside retinal imaging, electrophysiology, and childhood biometry to examine the contribution of long reads and the clinical diversity of cone-opsin disorders.

## 2. Results

### 2.1. Clinical Findings and Phenotypic Spectrum

**Figure 1:**
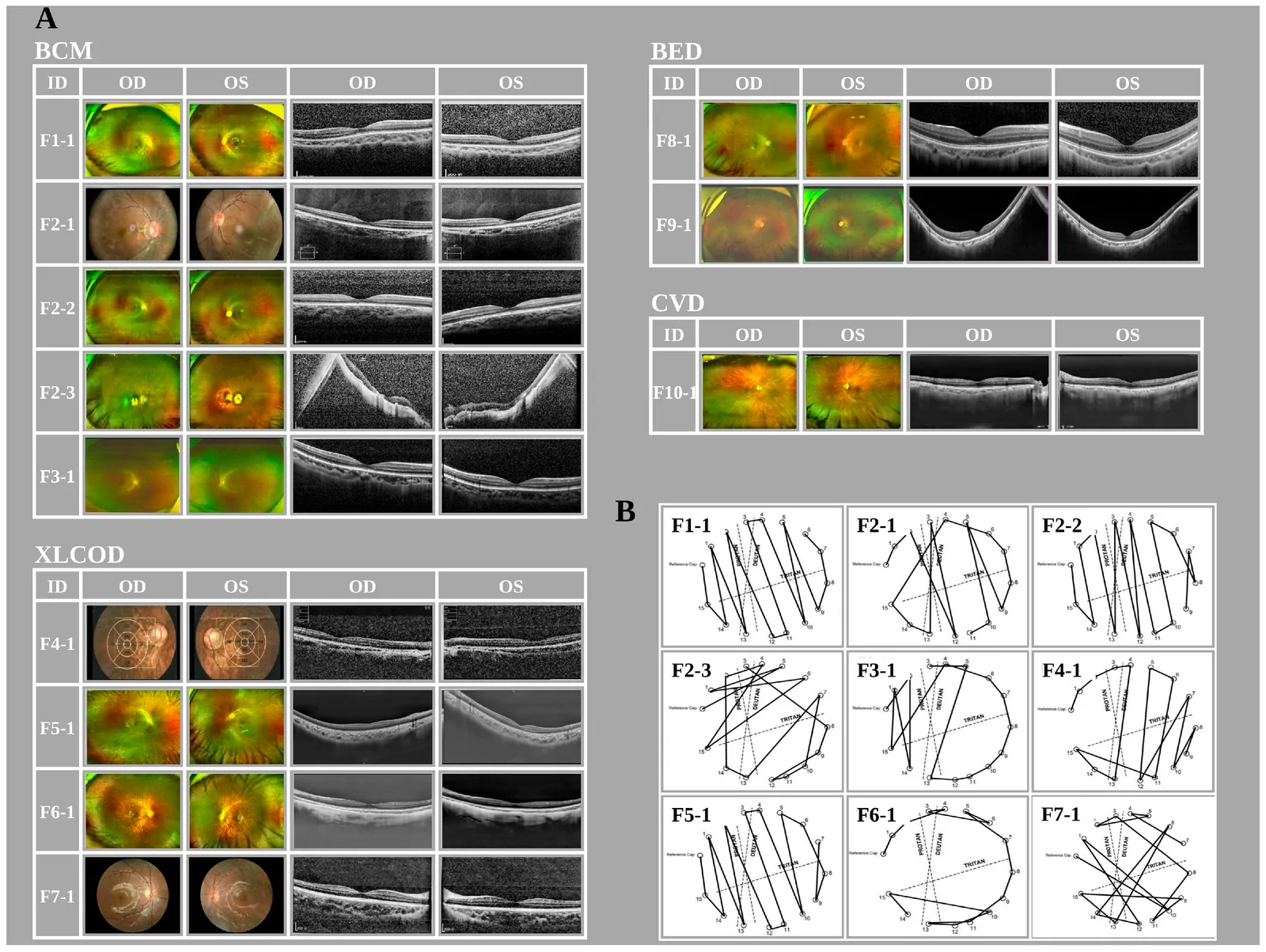
Multimodal retinal imaging and color vision in *OPN1LW*/*OPN1MW*-associated disorders. (**A**) Fundus photographs and optical coherence tomography (OCT) images from all 12 patients. Within each patient row, the first OD/OS pair contains fundus photographs, and the second pair contains OCT images; OD denotes the right eye and OS the left eye. Fundus autofluorescence (FAF) images from nine patients are provided in Supplementary Figure S1. Common findings included fundus tessellation, tilted optic discs, and focal ellipsoid zone/interdigitation zone (EZ/IZ) abnormalities. F2-3 had advanced macular atrophy, diffuse retinal thinning with outer retinal loss, and corresponding hypoautofluorescence. Clinical details are provided in Table 1. Fundus images for F4-1 were acquired with a Topcon 3D OCT system; those for F2-1 and F7-1 were obtained using Zeiss non-mydriatic fundus cameras; all remaining fundus photographs were acquired with Optos ultra-widefield imaging systems. (**B**) Farnsworth Panel D-15 results for nine patients. Most abnormal arrangements followed protan/deutan confusion axes. F2-3 and F7-1 had mixed or indeterminate patterns, whereas F6-1 had a borderline or near-normal result. Solid lines connect the color caps in the recorded arrangement, and dashed lines indicate the protan, deutan and tritan confusion axes. Crossings of the recorded paths and confusion axes are retained.

**Figure 2:**
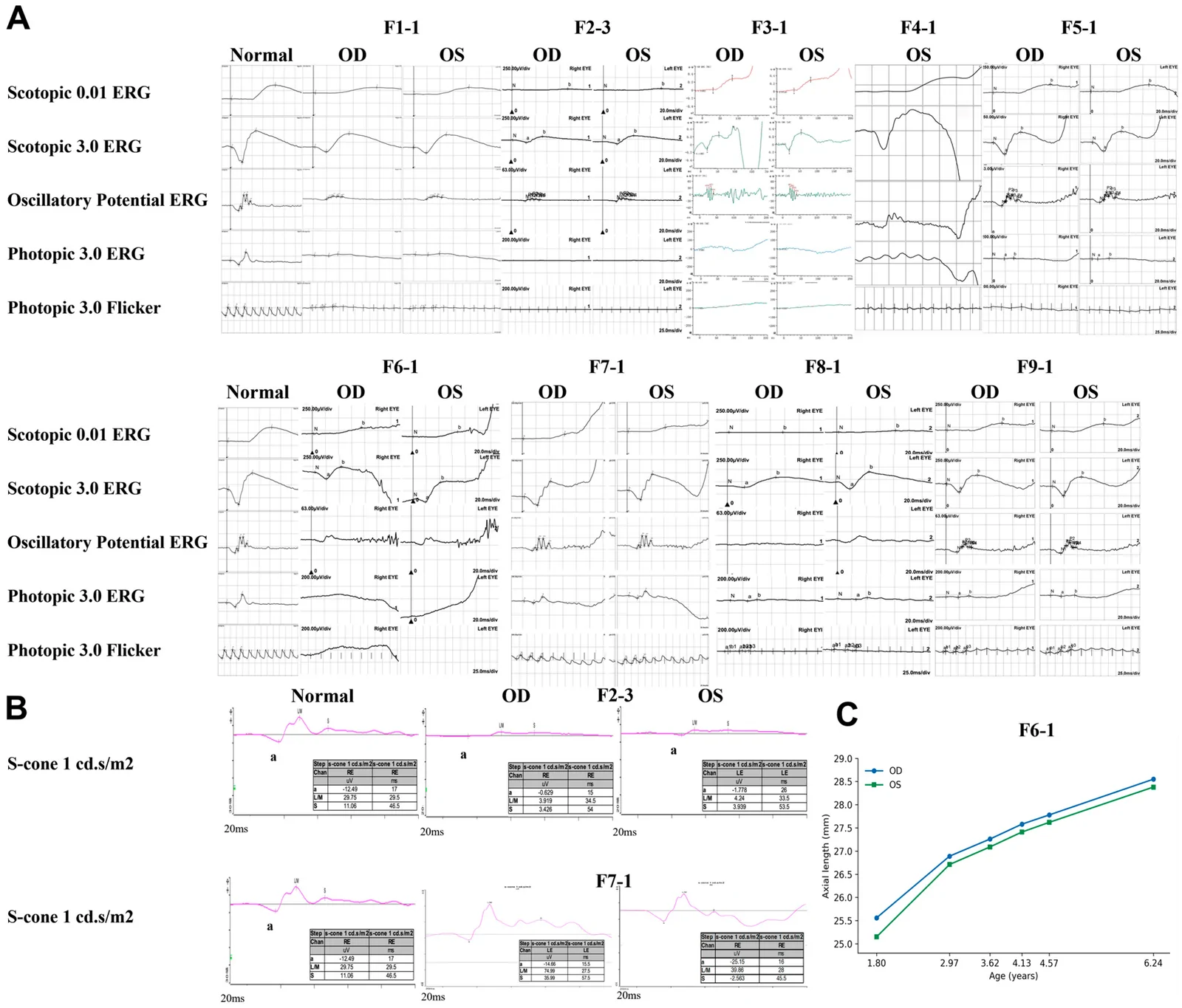
Electroretinographic findings and longitudinal axial length. (**A**) Full-field ERG recordings from nine patients, arranged in two rows with the displayed OD and OS labels and aligned response types. The normal reference column is repeated for comparison; it does not represent additional participants. Patients with BCM had markedly reduced or nearly extinguished photopic responses; F2-3 also had severely reduced rod responses. Photopic responses and preservation of dark-adapted function varied in XLCOD and BED. Only OS is shown for F4-1 because cooperation was insufficient for a reliable OD recording. Normal reference ERG traces for comparison were acquired from healthy control subjects with unremarkable ocular findings. These traces show well-preserved scotopic, photopic, and 30 Hz flicker responses, with amplitudes and implicit times falling within our laboratory established institutional reference ranges. The normal reference traces were not age-matched to individual patients. (**B**) S-cone recordings at the displayed flash strength of 1 cd·s/m^2^. F2-3 had identifiable but attenuated responses. Eye-specific interpretation of the F7-1 recordings is limited by inconsistent channel labels in the source records. Both patients’ recordings are descriptive because adherence to the extended S-cone protocol could not be verified. F7-1 is broadly consistent with normal results using our testing protocol. (**C**) Serial axial-length measurements for F6-1 at 1.80, 2.97, 3.62, 4.13, 4.57 and 6.24 years. Blue circles indicate OD, and green squares indicate OS. Axial length increased from 25.56 to 28.55 mm in OD and from 25.15 to 28.38 mm in OS between 1.80 and 6.24 years. All six measurements are listed in Supplementary Table S1.

Twelve male patients from 10 unrelated, non-consanguineous Chinese families were included. Family F2 contributed three patients, and every other family contributed one. Age at first visit ranged from 1 to 78 years, and recorded decimal visual acuity ranged from 0.01 to approximately 0.90. Clinical groups comprised BCM (*n* = 5), XLCOD (*n* = 4), BED (*n* = 2), and color vision deficiency (CVD; *n* = 1). Multimodal imaging is shown in Figure 1A. Fundus autofluorescence (FAF) images from nine patients are shown in Supplementary Figure S1. Farnsworth Panel D-15 results for nine patients are shown in Figure 1B, and full-field electroretinography (ERG) recordings for nine patients are shown in Figure 2A. Documented examination ages and the availability of imaging and electrophysiology are summarized in Table 1.

#### 2.1.1. Blue Cone Monochromacy

The BCM group comprised five patients (F1-1, F2-1, F2-2, F2-3, and F3-1). F1-1 had recorded visual acuity of 0.5 in both eyes, high myopic astigmatism recorded, partial failure on color-plate testing, and a protan/deutan confusion axis on D-15 testing. Fundus examination showed a tessellated fundus, mildly tilted optic discs, and peripapillary atrophy. Optical coherence tomography (OCT) and FAF were unremarkable (Supplementary Figure S1). Full-field ERG showed nearly extinguished or severely reduced cone responses and moderately reduced rod responses (Figure 2A).

The three affected members of Family F2 showed marked intrafamilial variation and shared the reported *OPN1LW* c.161dup variant. F2-1 and F2-2 had recorded visual acuity of 0.3 to 0.4, myopic astigmatism, axial lengths of 24.70 to 26.45 mm, protan/deutan color-confusion patterns, and focal foveal ellipsoid zone/interdigitation zone (EZ/IZ) abnormalities. F2-2 also underwent strabismus surgery at 5 years. Their grandfather, F2-3, had recorded visual acuity of 0.01 in the right eye and 0.05 in the left eye, high myopia, macular atrophy, and diffuse retinal thinning with outer retinal disruption. Corresponding hypoautofluorescence was present (Supplementary Figure S1). Rod responses were severely reduced, and photopic responses were extinguished (Figure 2A). S-cone responses remained identifiable but were reduced in amplitude (Figure 2B).

F3-1 had a severe BCM phenotype associated with an LCR-containing deletion. At 8 years, visual acuity was 0.15 in both eyes, with approximately −6.00 D of myopia and astigmatism. D-15 testing showed a protan/deutan confusion axis. Fundus examination and imaging showed a tilted optic disc, pigmentary and autofluorescence changes (Supplementary Figure S1), EZ disruption, and vitreous opacities. Dark-adapted responses remained recordable, whereas photopic 3.0 and 30 Hz responses were nearly extinguished.

#### 2.1.2. X-Linked Cone Dystrophy

Four patients (F4-1, F5-1, F6-1, and F7-1) were classified as XLCOD, with substantial variation in refraction, retinal structure, and ERG findings (Figure 1A and Figure 2A). F4-1 had visual acuity of 0.2 in both eyes, myopic astigmatism, axial lengths of 26.25 and 25.96 mm, and a protan/deutan D-15 pattern. Fundus examination showed tessellation and peripapillary atrophy; OCT showed EZ disruption, and photopic responses were markedly reduced or nearly extinguished. F5-1 had visual acuity of approximately 0.3 in the right eye and 0.2 in the left eye, hyperopic astigmatism, and axial lengths of 24.20 and 24.15 mm. Color-plate performance was poor, with a protan/deutan confusion axis on D-15 testing. OCT showed EZ blurring. Dark-adapted responses were largely preserved, whereas photopic and 30 Hz responses were moderately reduced.

F6-1 had an XLCOD phenotype with severe early-onset myopia. Recorded visual acuity was 0.3 in both eyes, with severe myopic astigmatism. Axial lengths were 25.56 mm in the right eye and 25.15 mm in the left eye at 1.80 years, increasing to 28.55 and 28.38 mm, respectively, at 6.24 years. Color-plate testing was partly abnormal, but D-15 performance was borderline or near normal, without a definite confusion axis. Fundus examination showed tessellation and tilted optic discs. OCT showed foveal IZ disruption, and cone responses were markedly reduced.

F7-1 had milder structural findings, recorded visual acuity of 0.6 in both eyes at 8 years, mild hyperopic astigmatism, and axial lengths of 23.68 and 24.59 mm at 7.54 years, measured at an earlier visit than the refraction. Color testing showed impaired blue recognition and a mixed or indeterminate D-15 arrangement. The foveal reflex was reduced, but OCT and FAF were unremarkable (Supplementary Figure S1). Cone responses were reduced. S-cone recordings were available (Figure 2B), but inconsistent channel labels and incomplete protocol documentation precluded a conclusion that S-cone function was normal.

#### 2.1.3. Bornholm Eye Disease

F8-1 and F9-1 were classified as BED (Figure 1A and Figure 2A). F8-1 had recorded visual acuity of approximately 0.9 in the right eye and 0.7 in the left eye, approximately −7.00 D of myopia with astigmatism, and axial lengths of 27.56 and 27.78 mm measured at 8 years. Color-plate testing showed a marked red–green defect. OCT showed foveal IZ disruption, dark-adapted ERG responses were normal, and photopic responses were mildly to moderately reduced. F9-1 had visual acuity of 0.5 in the right eye and 0.6 in the left eye, approximately −8.50 D of myopia with astigmatism, and axial lengths exceeding 27 mm at both documented examinations. Fundus imaging showed tilted optic discs, peripapillary atrophy, and tessellation. OCT showed posterior staphyloma with foveal EZ/IZ blurring, and FAF showed extensive hypoautofluorescence at the posterior pole (Supplementary Figure S1). Dark-adapted responses were normal, whereas photopic responses were moderately reduced. Both patients had high myopia, cone dysfunction, red–green color vision impairment, and relative preservation of rod-mediated function.

#### 2.1.4. Color Vision Deficiency

F10-1 was the only patient in the CVD group. At 78 years, visual acuity was 0.4 in both eyes, with mild hyperopic astigmatism. Pseudoisochromatic plate testing showed partial failure and a red–green defect. Fundus examination showed tessellation and temporal optic disc pallor. OCT showed focal foveal IZ disruption, and cataract was present (Figure 1A; Table 1). Three grandsons reportedly had a similar color vision phenotype. Full-field ERG was not recorded, so this descriptive classification does not establish normal cone function.

### 2.2. Axial Length and Longitudinal Change

Axial-length measurements retained for analysis were available for eight of the 12 patients (Table 1), with values ranging from 23.68 to 28.55 mm. In F6-1, axial length increased from 25.56 to 28.55 mm in OD and from 25.15 to 28.38 mm in OS between 1.80 and 6.24 years (Figure 2C; Supplementary Table S1). F8-1 had axial lengths of 27.56 and 27.78 mm at 8.36 years. In F9-1, axial lengths were 27.47 mm (OD) and 27.30 mm (OS) at 7.34 years, and 27.70 mm (OD) and 27.36 mm (OS) at 10.00 years; this small increase does not establish a longitudinal growth rate.

F1-1 was excluded from axial-length analysis because the recorded biometry was considered unreliable after review and is shown as NR in Table 1. F5-1 and F7-1 had axial lengths of 24.20/24.15 mm and 23.68/24.59 mm, respectively, and values in F2-1, F2-2, and F4-1 ranged from 24.70 to 26.45 mm. Differences in examination age and follow-up precluded estimation of a cohort-level elongation rate. Corneal and lenticular contributions to refractive error were not systematically assessed, and keratometry was not available for F1-1.

F6-1 provides the principal serial observation (Figure 2C; Supplementary Table S1). Across six dated records from 19 October 2020 to 31 March 2025 (ages 1.80 to 6.24 years), the mean axial length across both eyes increased from 25.36 to 28.47 mm. The endpoint difference was 3.11 mm over 1624 days (4.45 years), approximately 0.70 mm/year; this is a descriptive first-to-last estimate, not a regression. Two historical axial-length observations were recorded for F2-2: 24.70 mm in OD and 24.79 mm in OS at a reported age of 6.00 years, and 25.20 mm in OD and 25.28 mm in OS at a reported age of 8.00 years; these were insufficient to define a robust trajectory, as were the two F9-1 measurements. Sustained axial elongation is therefore reported as an individual finding in F6-1.

### 2.3. Genetic Findings

**Figure 3:**
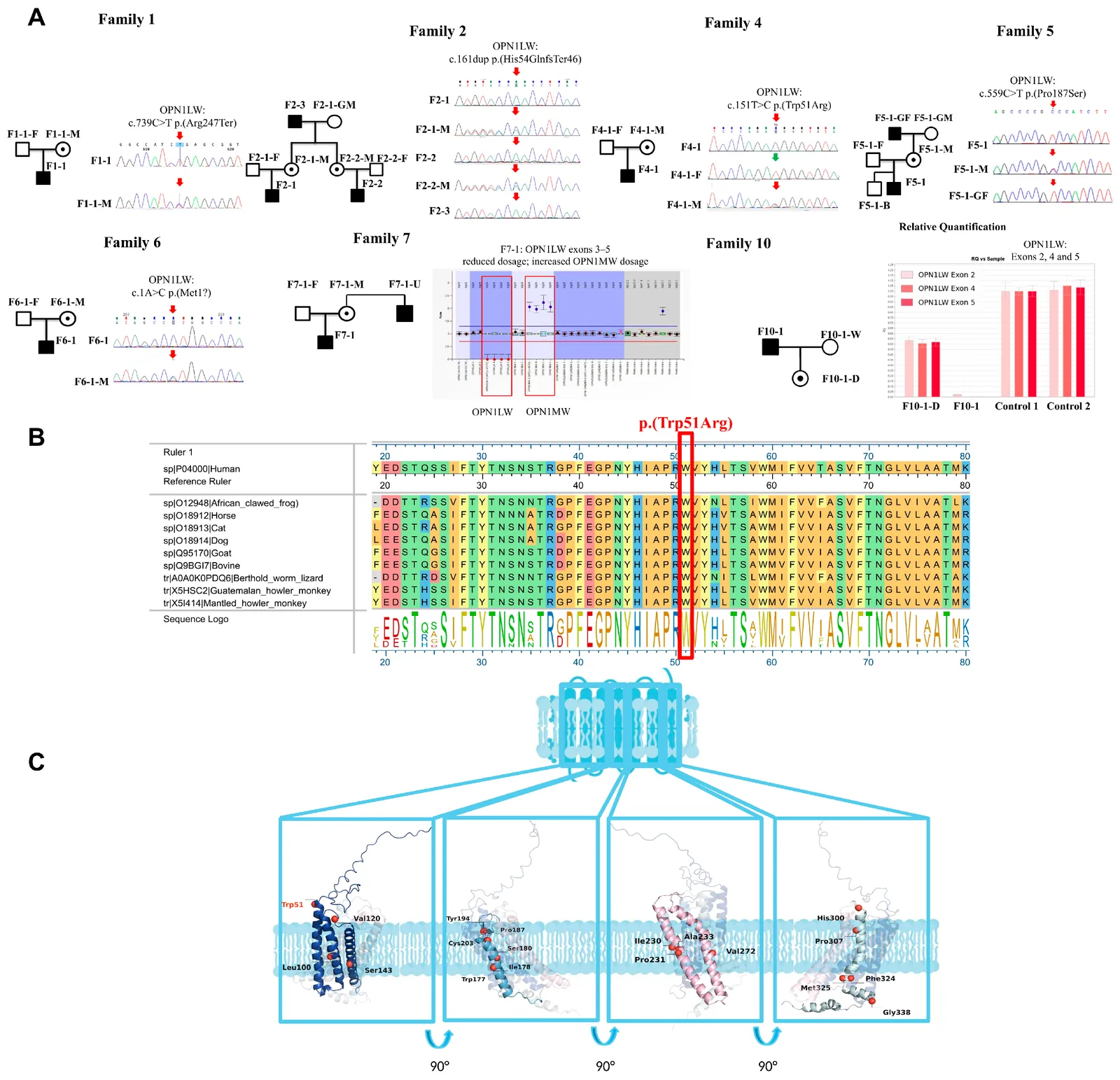
Sequence variants, family evidence and protein analyses. (**A**) Pedigrees and available Sanger panels for Families 1, 2, 4, 5 and 6, with family dosage panels for Families 7 and 10. Squares denote males, circles females, and filled symbols affected individuals; a central dot denotes a heterozygous carrier confirmed by testing. Arrows identify the nucleotide position of interest (red, variant present; green, reference sequence). Suffixes F, M, GF, GM, B, U, D and W denote father, mother, grandfather, grandmother, brother, uncle, daughter and wife, respectively. Additional tested relatives are not counted as cohort patients. In Family 5, the variant was detected in the proband, his mother and maternal grandfather and was absent in his older brother (F5-1-B); ocular symptoms were reported for the grandfather. The Family 7 MLPA result displayed the reported reduction in *OPN1LW* exon 3–5 dosage and increase in *OPN1MW* exon 3–5 dosage. The Family 10 qPCR plot shows *OPN1LW* exons 2, 4 and 5 for F10-1, his daughter (F10-1-D) and two controls; no L-specific signal was detected in F10-1, and his daughter showed about half of the control dosage. (**B**) Alignment of the L-opsin region surrounding residue 51 in ten vertebrate species, with human UniProt P04000 at the top. The highlighted column and sequence logo show conservation of Trp51. The red rectangle marks residue 51. (**C**) Predicted L-opsin structure relative to a membrane schematic. Four expanded views are rotated by 90° about the vertical axis. The blue schematic represents the membrane; cyan outlines and connecting lines link the overview to the enlarged structural views, and curved arrows indicate successive 90° rotations. The labeled residues mark reported missense-variant sites, and red spheres indicate their positions in the structural model. Trp51 and Pro187 are the sites of the cohort missense variants p.(Trp51Arg) and p.(Pro187Ser), respectively; the other labeled sites were obtained from the Human Gene Mutation Database (HGMD) [48]. Trp51 is labeled in red and the other residues in black. Arg247 is not shown because the cohort variant p.(Arg247Ter) is a nonsense variant. These positional annotations provide structural context and do not establish functional effects or pathogenicity. Protein-variant labels use HGVS three-letter notation, consistent with Table 3.

A summary of the molecular assays performed, corresponding variant findings, and the basis for each interpretation across all patients is provided in Table 2; patient-level details, including genomic coordinates in GRCh38 and GRCh37, are given in Supplementary Table S2. Pedigrees and Sanger sequencing results for Families 1, 2, 4, 5, and 6, together with pedigrees and dosage results for Families 7 and 10, are shown in Figure 3A. All five sequence variants were confirmed by Sanger sequencing of *OPN1LW*-specific amplicons; in F1-1, the trace containing c.739C>T also showed L-type bases at exon 4 codons 230, 233 and 236. ACMG/AMP classification of the five sequence variants is presented in Table 3. For the *OPN1LW* c.1A>C (p.Met1?) variant, a downstream ATG located at +55 bp relative to the canonical start codon was assessed. Translation initiated from this downstream ATG would result in a frameshift followed by a premature termination codon after 42 amino acids, thereby eliminating production of a functional full-length protein rather than partially preserving activity. In F1-1 and Family F2, a single truncating *OPN1LW* variant was identified, but the array composition, and hence the precise mechanism for loss of both L- and M-cone function in BCM, was not determined. Multiplex ligation-dependent probe amplification (MLPA) in F7-1 identified a dosage decrease in *OPN1LW* exons 3 to 5 with a corresponding increase in *OPN1MW* copy number, suggesting a possible hybrid gene structure. In F10-1, qPCR detected no *OPN1LW*-specific signal at exons 2, 4 and 5, whereas his daughter showed about half of the control dosage. Because these assays detect L-specific sequences, the patterns in Families F7 and F10 are compatible with a possible hybrid gene; the complete array structure was not resolved. The F3-1 deletion and these dosage findings were not formally classified under the ACMG/ClinGen CNV framework [19].

Computational predictions for *OPN1LW* p.(Trp51Arg) were discordant (Supplementary Table S3) [20,21,22,23,24,25,26,27,28,29,30,31,32,33,34,35,36,37,38,39,40,41,42,43]. The REVEL score of 0.417 falls within the interval that supports neither PP3 nor BP4 under the calibration of Pejaver et al. [23]. The variant is classified as a VUS in this study; computational predictions do not establish its functional effect. Sequence conservation at Trp51 and its location in the structural model are shown in Figure 3B,C [44,45,46].

**Table 2:** Molecular assays, variant findings, and interpretation in the cohort.

Patient	Molecular Assays Performed	Molecular Finding	Basis of Interpretation
F1-1	WES; Sanger	*OPN1LW* c.739C>T p.(Arg247Ter); Likely pathogenic	Confirmed by *OPN1LW*-specific Sanger sequencing, with L-type bases at exon 4 codons 230, 233 and 236; array configuration not determined.
F2-1 F2-2 F2-3	WES; Sanger	*OPN1LW* c.161dup p.(His54GlnfsTer46); Pathogenic	Confirmed by *OPN1LW*-specific Sanger sequencing; co-segregation in three affected relatives; array configuration not determined.
F3-1	WES; WGS; ONT; junction PCR/Sanger	LCR-containing deletion chrX:g.154127837_154179624del (hg38; 51,788 bp) chrX:g.153393306_153445112del (hg19; 51,807 bp)	Breakpoint-junction evidence was obtained from long-read sequencing and confirmed by Sanger sequencing of the junction PCR product; the amplicon was detected in the proband and his mother, but not in his unaffected father.
F4-1	WES; Sanger	*OPN1LW* c.151T>C p.(Trp51Arg); VUS	Confirmed by *OPN1LW*-specific Sanger sequencing; array configuration not determined.
F5-1	WES; Sanger	*OPN1LW* c.559C>T p.(Pro187Ser); Likely pathogenic	Confirmed by *OPN1LW*-specific Sanger sequencing; array configuration not determined.
F6-1	WES; Sanger	*OPN1LW* c.1A>C p.(Met1?); Likely pathogenic	Confirmed by *OPN1LW*-specific Sanger sequencing; array configuration not determined.
F7-1	WES; MLPA	*OPN1LW* exon 3–5 dosage decrease; increased *OPN1MW* dosage	Dosage patterns suggest a structural rearrangement, possibly a hybrid gene.
F8-1	WES; WGS; ONT; Sanger	OPN1LW-LVAVA/OPN1MW-MVAVA Exon 3 haplotypes	Long-read sequencing resolved the proband’s haplotypes; the maternal *OPN1LW* allele was confirmed by Sanger, whereas the maternal *OPN1MW* haplotype was inferred from WES and not fully confirmed.
F9-1	WES; WGS; ONT	OPN1LW-LIAVA/OPN1MW-MVVVA Exon 3 haplotypes	Long-read sequencing resolved haplotypes that short-read sequencing could not reliably dissect. Mother not tested.
F10-1	WES; qPCR	*OPN1LW* exon 2–5 deletion (inferred)	The *OPN1LW* exon 2–5 deletion inferred from WES data was assessed by qPCR at exons 2, 4 and 5: no L-specific signal was detected in F10-1, and his daughter showed about half of the control dosage. A hybrid gene cannot be excluded; not formally classified.

Reference transcripts: *OPN1LW* NM_020061.6; *OPN1MW* NM_000513.2.

**Table 3:** ACMG/AMP classification of *OPN1LW* sequence variants identified in this study.

cDNA Change	Protein Change	Variant Type	GnomAD Frequency	ClinVar/Literature Status	Criteria Applied	ACMG/AMP Classification ^1^
c.739C>T	p.(Arg247Ter)	Nonsense	Absent	ClinVar: Pathogenic (ID 10503; single submission)	PVS1, PM2	Likely pathogenic
c.161dup	p.(His54GlnfsTer46)	Frameshift	Absent	No ClinVar submission	PVS1, PM2, PP1	Pathogenic
c.151T>C	p.(Trp51Arg)	Missense	Absent	No ClinVar submission	PM2	Uncertain significance (VUS)
c.559C>T	p.(Pro187Ser)	Missense	Absent	Functional study: Sechrest et al., 2024 [47]	PM2, PP3, PS3	Likely pathogenic
c.1A>C	p.(Met1?)	Start-loss	Absent	No ClinVar submission	PVS1, PM2	Likely pathogenic

*OPN1LW* transcript: NM_020061.6. ^1^ According to the 2015 ACMG/AMP standards and guidelines for sequence-variant interpretation. PS3 for p.(Pro187Ser) refers to well-established functional studies demonstrating a damaging effect: human OPN1LW-p.Pro187Ser expressed via AAV in Opn1mw-knockout mouse cones showed reduced protein expression and abnormal ERG responses [47].

### 2.4. Long-Read Analysis of Exon 3 Haplotypes and Structural Variation

**Figure 4:**
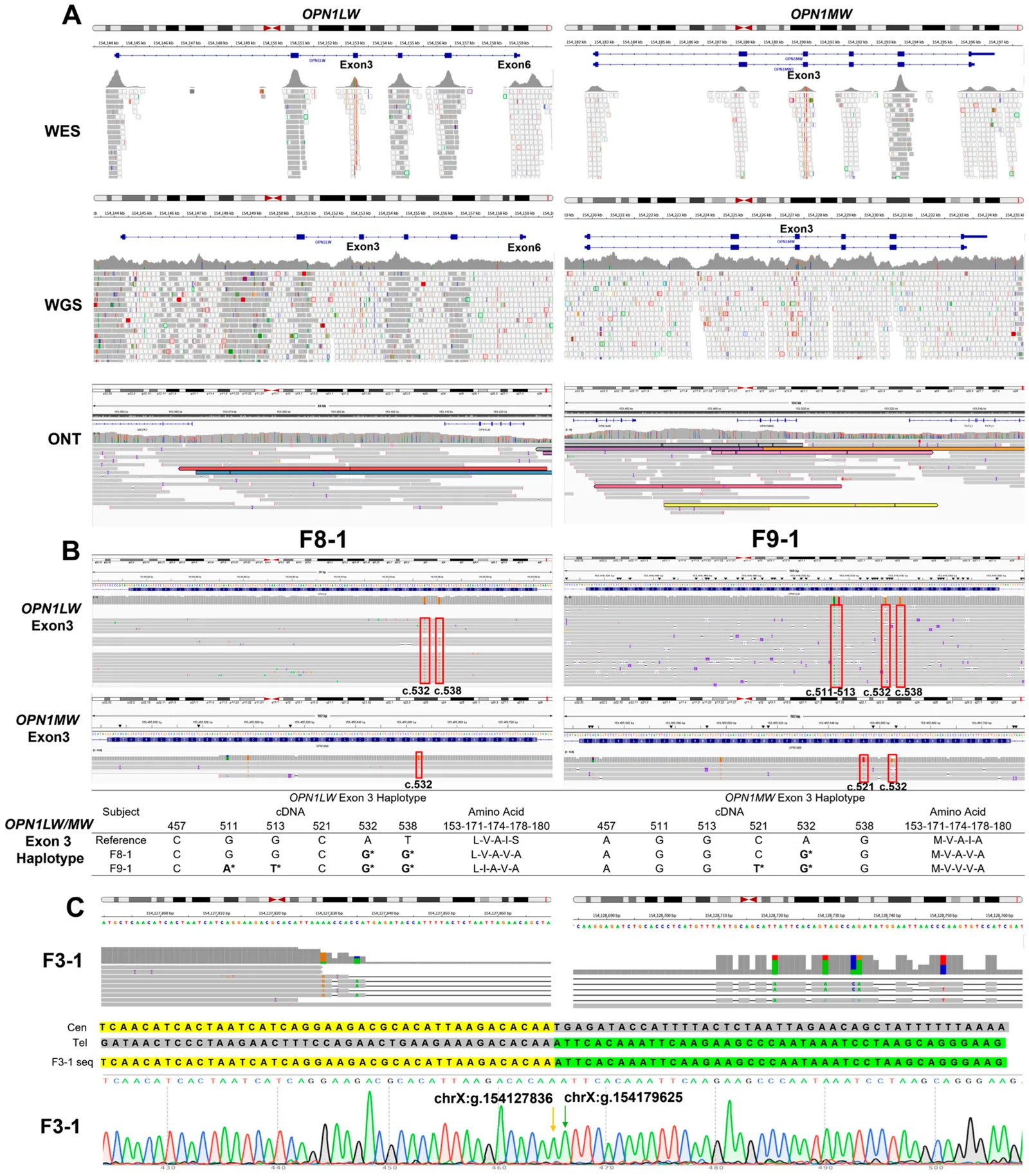
Long-read analysis of the *OPN1LW*/*OPN1MW* gene cluster. (**A**) Integrative Genomics Viewer (IGV) alignments for WES, WGS and ONT, with *OPN1LW* and *OPN1MW* column labels. Low-mapping-quality short reads illustrate assignment difficulty at this homologous locus. Long reads provide additional alignment evidence without establishing complete array resolution in every patient. (**B**) Exon 3 alignments in F8-1 and F9-1, with the displayed nucleotide combinations and amino-acid haplotypes. Asterisks (*) indicate differences from the displayed reference. F8-1 carries *OPN1LW*-LVAVA with *OPN1MW*-MVAVA; F9-1 carries *OPN1LW*-LIAVA with *OPN1MW*-MVVVA. (**C**) ONT-based delineation of the structural variant chrX:g.154127837_154179624del (hg38) in F3-1. IGV snapshots illustrate the breakpoint locations on the centromeric and putative telomeric sides. Although reads are visible at the *OPN1LW* locus, comparative alignment analysis shows that they carry *OPN1MW*-specific SNPs, particularly in exon 5, confirming that the entire *OPN1LW* gene is deleted and that the telomeric breakpoint lies between *OPN1LW* and *OPN1MW*. Sanger sequencing of the breakpoint-spanning amplicon confirmed the direct joining of chrX:g.154127836 and chrX:g.154179625 in F3-1. Yellow and green shading represent sequences retained from the centromeric and telomeric sides, and gray shading marks the deleted region.

In F3-1, F8-1, and F9-1, short-read sequencing did not establish a molecular diagnosis, prompting Oxford Nanopore sequencing of five individuals: F3-1, F8-1 and F9-1, together with the fathers F3-1F and F9-1F (Figure 4A; Supplementary Table S4). To directly assess the performance of short-read sequencing across the *OPN1LW*/*OPN1MW* cluster, we quantified uniquely mapped (MAPQ ≥ 20) coverage from WES and WGS data of two families (F8 and F9), in whom ONT sequencing identified gene recombination at this locus. Despite satisfactory overall short-read quality (Supplementary Table S4), uniquely mapped coverage dropped markedly within the high-homology opsin exons: 0.00% of bases achieved ≥20× uniquely mapped depth in *OPN1LW* exon 3 and *OPN1MW* exons 1, 3 and 4 across all samples. Control regions outside this gene cluster remained unaffected, confirming that this coverage loss is specific to the high-sequence homology between L and M opsin genes rather than a general defect in sequencing quality (Supplementary Table S5). Gene identity (L vs. M) was assigned from ONT reads spanning exon 3 to exon 5 of the opsin array; the number of such informative reads in each sample is given in Supplementary Table S4.

F8-1 carried the *OPN1LW*-LVAVA and *OPN1MW*-MVAVA haplotypes. Sanger sequencing with *OPN1LW*-specific primers confirmed the maternal *OPN1LW* allele, whereas the maternal *OPN1MW* haplotype was inferred from WES and was not fully confirmed. F9-1 carried *OPN1LW*-LIAVA and *OPN1MW*-MVVVA (Figure 4B; Supplementary Figure S2). Family histories are summarized in Table 1; myopia was reported in the mother of F8-1, but a comparable cone disease phenotype was not documented in either mother. Published associations of LIAVA and LVAVA with abnormal exon 3 splicing and myopia provide context [7,15,16,17,18]. The accompanying *OPN1MW* haplotypes were not independently shown to be pathogenic, and complete copy-specific array resolution should not be assumed for every patient. Exon 3 haplotypes were not systematically assessed in the remaining patients with other molecular findings.

In F3-1, Oxford Nanopore sequencing identified an approximately 51.8 kb LCR-containing maternally inherited structural deletion: chrX: g.154127837_154179624del (hg38; 51,788 bp), corresponding to chrX: g.153393306_153445112del (hg19; 51,807 bp) (Figure 4C; Supplementary Figure S2A). The upstream breakpoint lies upstream of the LCR, and the downstream breakpoint lies between *OPN1LW* and *OPN1MW*; therefore, the entire LCR and *OPN1LW* are deleted, while *OPN1MW* and the downstream sequence of the array remain. LCR involvement is consistent with an established mechanism of BCM [5,6,10]. Sequence alignment revealed a 9 bp microhomology (AAGACACAA) present at both breakpoint regions, with only a single copy retained in the rearranged allele (Figure 4C). Although this breakpoint-flanking microhomology alone cannot definitively confirm the underlying recombination mechanism, it supports microhomology-mediated end joining (MMEJ) as a plausible mechanistic hypothesis for this rearrangement.

## 3. Discussion

Previous studies have described a broad clinical spectrum associated with *OPN1LW*/*OPN1MW* variants [5,7,10,11,12,13,14,15,16,17,18]. Our integrated molecular and ophthalmic assessment illustrates this heterogeneity. BCM was associated with severe cone dysfunction and variable rod involvement, whereas XLCOD showed variable refractive and electrophysiological findings. BED was characterized by early high myopia and relative preservation of rod function. Given the clinical overlap among these entities, molecular confirmation of pathogenic *OPN1LW*/*OPN1MW* variants provides the basis for classification, as clinical findings alone may not reliably distinguish milder cases.

LCR and opsin-array disruption are established causes of BCM [5,6,10]. In F3-1, long-read sequencing helped characterize an LCR-containing deletion that remained unresolved after short-read analysis. This illustrates how long reads can contribute to analysis of highly homologous arrays [8,9].

The shared c.161dup finding in Family F2 accompanied marked differences between generations. F2-3 had macular atrophy at 59 years, whereas his young grandsons had more limited foveal abnormalities. Carroll et al. reported heterogeneous retinal structure, residual parafoveal cones, and progressive visual loss in some cone-opsin phenotypes [49]. Patterson et al. visualized residual cone inner segments and found no significant structural decline during the available short follow-up: OCT over 12 months and AOSLO for up to 18 months [50]. Those observations neither establish lifelong stability nor show a uniform degenerative course. Impaired cone function should not be equated with complete cone loss. Age-related progression may contribute to the differences in Family F2, but myopia-related pathology and other ocular factors may also contribute. This cross-sectional comparison cannot predict either grandson’s future phenotype or quantify an individual degeneration rate.

The p.(Pro187Ser) substitution requires gene-specific interpretation. An earlier clinical report involved an *OPN1MW*-containing array, whereas Sechrest et al. tested a human OPN1LW P187S construct delivered by AAV to *Opn1mw*-knockout mouse cones [47,51]. The study found markedly reduced detectable mutant protein and no restored L/M-cone light response. This published functional evidence is relevant to the study assessment of likely pathogenicity, but it is not a new patient-derived experiment and does not establish F5-1′s complete gene-array structure or endogenous transcript processing [52]. F5-1 was investigated by family-based WES, and Sanger sequencing covered the proband, his mother, maternal grandfather and older brother. The variant was present in the proband, mother and maternal grandfather and absent in the older brother; ocular symptoms were reported for the grandfather. These relatives were not evaluated as a standardized clinical segregation series, so the family findings are interpreted descriptively.

The sequence-variant classifications reported in Table 3 are distinct from the completeness of molecular resolution. The LCR-containing deletion is consistent with an established disease mechanism, whereas exon-dosage patterns and exon 3 haplotypes require interpretation in relation to allele structure and expressed copies. An *OPN1LW* finding alone should not be assumed to explain loss of both L- and M-cone function without corresponding array and phenotypic evidence [6,9,19,53].

A tiered diagnostic approach is useful for this locus. Short-read sequencing and Sanger validation can identify sequence variants, while MLPA or comparable assays can assess exon dosage. Long reads can help investigate unresolved array order, hybrid genes, LCR involvement, and complex rearrangements. In this study, long reads helped clarify selected findings that remained ambiguous after conventional testing. The first two genes downstream of the LCR are generally considered the main expressed copies, making array position important for variant interpretation [3,4,6,8,9].

Exon 3 splicing defects and residual cone structure vary among cone-opsin disorders [7,14,15,16,17,18,50]. In 1.226 Chinese families with early-onset high myopia, Wang et al. identified disease-associated *OPN1LW* haplotypes or truncating variants in 68 families [54]. *OPN1LW*-LVAVA/LIVVA were associated with high myopia without protanopia, whereas *OPN1LW*-LIAVA and LVAVA in an L–M hybrid gene were associated with high myopia and protanopia. These findings provide a relevant comparison for F8-1 and F9-1, who had high myopia and distinct reported *OPN1LW*-LVAVA/LIAVA haplotypes. However, their red–green defects were not fully subtyped, and their complete array configurations were not established. The haplotype labels alone cannot assign them to the published subgroups. The accompanying *OPN1MW* haplotypes should be interpreted separately, with attention to gene-copy identity and array position.

Serial biometry in F6-1 documented continued axial elongation over 4.45 years. Axial lengths were already long at his first recorded visit, but earlier measurements were unavailable. F7-1 had shorter recorded axial lengths, illustrating variation between patients; the measurements were obtained at different examination ages. During normal emmetropization, axial growth is coordinated with changes in corneal and lenticular power [55]. The observations in F6-1 suggest early disruption of ocular growth, but they cannot distinguish prenatal differences from rapid postnatal elongation. Limited longitudinal data in the other patients preclude a general conclusion about progression.

Variation in exon 3 splicing and cone structure provides a biological context for altered retinal signaling [7,15,17,18,50,56]. Neitz et al. proposed that differences in photopigment content among neighboring cones generate spurious contrast signals through retinal center–surround circuitry, potentially promoting ocular elongation [57]. This hypothesis is relevant to altered emmetropization in opsin-related disease, but local contrast signaling was not measured in our cohort. F6-1 had long axial lengths despite borderline or near-normal D-15 performance. These observations do not establish the functional effect of c.1A>C on cone input or prove a causal mechanism for axial elongation. Experimental studies implicate choroidal and scleral remodeling in ocular enlargement [58,59]. These processes offer a possible downstream context for altered retinal input. We did not measure choroidal thickness, scleral biomechanics, or local retinal signaling, so the present observations do not establish this proposed pathway.

This small retrospective cohort, non-uniform testing and follow-up, incomplete S-cone protocol documentation, and unresolved copy-specific contributions limit inference. ONT was used primarily after an unresolved conventional work-up rather than uniformly across the cohort, introducing selection bias and preventing an unbiased comparison of diagnostic methods. Subgroup assignment was partly genotype-informed and was supported by limited evidence. Long reads are especially useful for LCR-containing structural variants, while exon 3 haplotypes and residual cone structure require joint interpretation. Future studies should combine early standardized biometry, color testing, ERG, OCT/FAF, and phased gene-array analysis, alongside multi-locus interaction analyses to separate opsin effects from other myopia susceptibility loci. The present findings support integrated molecular assessment and early ocular-growth surveillance without establishing a single mechanism for all cone-opsin disorders.

## 4. Materials and Methods

### 4.1. Study Cohort

This retrospective study included 12 male patients from ten unrelated Chinese families with clinically suspected X-linked cone-opsin disorders. Molecular findings in the *OPN1LW*/*OPN1MW* cluster included sequence variants, dosage abnormalities, an LCR-containing deletion, and exon 3 haplotypes. All participants were evaluated at the Department of Ophthalmology, Peking Union Medical College Hospital (PUMCH), Beijing, China, between 2013 and 2026. Cases were identified through clinical referrals and genetic testing requests, rather than consecutive or population-based ascertainment. Available clinical records and genetic results were reviewed, including earlier archived examinations where relevant. Samples from available relatives were used for segregation analysis. The study was approved by the Ethics Committee of Peking Union Medical College Hospital (approval No. JS-2059; 10 January 2019), which covered the retrospective use of clinical records, including those of patients evaluated before 2019; genetic testing and enrollment were performed after approval. The study followed the Declaration of Helsinki and the Guidance on Sample Collection of Human Genetic Diseases issued by the Ministry of Public Health of China. Written informed consent was obtained from participants or, for minors, their parents or legal guardians after the nature and possible consequences of the study had been explained.

### 4.2. Clinical Evaluations

Available clinical assessments included ocular and family histories, visual acuity recorded in decimal format, refraction, slit-lamp examination, axial-length measurement, dilated fundus examination, fundus photography, fundus autofluorescence (FAF), optical coherence tomography (OCT), color testing, and full-field electroretinography (ERG). Retinal imaging was performed using Topcon fundus/OCT systems (Topcon, Tokyo, Japan), Optos ultra-widefield imaging systems (Optos plc, Dunfermline, UK), Spectralis OCT systems (Heidelberg Engineering, Heidelberg, Germany), Zeiss non-mydriatic fundus cameras (Carl Zeiss Meditec AG, Jena, Germany), and the VG200 SS-OCT system (SVision Imaging, Ltd., Luoyang, China). When documented, histories included symptom onset, photophobia, visual behavior, and color discrimination. Visual-acuity measurements were abstracted from the clinical records in decimal notation, with unaided or corrected status retained as documented; in Chinese clinical practice, distance visual acuity is typically measured at 5 m with the standard logarithmic visual acuity chart (GB 11533-2011) [60]. F6-1 could not cooperate with quantitative acuity testing at 1.80 years, and a formal infant-acuity test was not documented. Cycloplegic refraction with atropine ophthalmic gel was documented for F6-1 on 24 December 2021; cycloplegic status was not consistently documented for the other refractions. Four intermediate axial-length examinations in F6-1 were performed using an IOLMaster 700 (Carl Zeiss Meditec AG, Jena, Germany); the devices for the first and last recorded values were not documented.

Color vision was assessed using pseudoisochromatic plates and/or the Farnsworth Panel D-15 test. Full-field ERGs were recorded with Diagnosys Espion E2 (Diagnosys LLC, Lowell, MA, USA) or RetiPort (Roland Consult Stasche & Finger GmbH, Brandenburg an der Havel, Germany) systems using ERG-jet or DTL electrodes, following the ISCEV Standard [61]. Available recordings included dark-adapted (DA) rod and combined responses, oscillatory potentials, light-adapted (LA) responses, and 30 Hz flicker. S-cone ERG recordings were available for F2-3 and F7-1 and were compared descriptively with normal traces obtained under the same institutional recording conditions. The recordings specified a flash strength of 1 cd·s/m^2^. Complete stimulus spectra, background illumination, and adaptation parameters were not documented, so adherence to the separate ISCEV S-cone extended protocol could not be verified [62]. S-cone amplitudes and implicit times were considered exploratory where identifiable. Patient S-cone waveforms were compared against internal normal controls recorded under the same laboratory conditions at our institution.

Clinical groups were assigned descriptively from the combined clinical, imaging, electrophysiological, and molecular findings. BCM is defined by early-onset severe color vision deficiency and cone dysfunction with relative preservation of rod and S-cone function, associated with pathogenic or likely pathogenic *OPN1LW* missense, nonsense, or frameshift mutations, structural variants involving the LCR or other variants. BED described early high myopia with red–green color vision deficits and associated exon 3 haplotypes, without the full severe BCM phenotype. XLCOD was assigned to patients with substantial cone dysfunction on ERG who did not meet the descriptive criteria for BCM or BED. CVD denoted the reported color-vision phenotype in F10-1; the absence of a recorded ERG prevents confirmation that cone function was otherwise normal. Subgroup assignment was partly genotype-informed with limited evidence: F2-1 and F2-2 had no recorded ffERG, S-cone ERG within the BCM group was available only for F2-3, and F1-1 and F2-3 had reduced rod responses. The BCM and BED labels are descriptive clinical classifications based on the available evidence.

### 4.3. Next-Generation Sequencing and Variant Interpretation

Genomic DNA was extracted from peripheral blood using the QIAamp DNA Blood Midi Kit (Qiagen, Hilden, Germany) and assessed with a NanoDrop spectrophotometer (Thermo Fisher Scientific, Waltham, MA, USA). The WES workflow used the xGen Exome Research Panel v2 (Integrated DNA Technologies, Coralville, IA, USA), an Illumina HiSeq platform (Illumina, San Diego, CA, USA), GRCh37 (hg19) alignment and NextGene v2.3.4 (SoftGenetics, State College, PA, USA). Trio WGS was performed in Families F3, F8 and F9 when initial testing did not resolve the opsin locus. Sample-level sequencing metrics are provided in Supplementary Table S3. Mapping-quality-filtered depth for *OPN1LW*/*OPN1MW* exons and control loci is summarized in Supplementary Table S4.

Population-frequency and annotation data from the 1000 Genomes Project, dbSNP, the Exome Aggregation Consortium, and gnomAD were used, when available, to filter common variants [63,64,65,66]. Variants in genes associated with inherited retinal disease, high myopia, color vision deficiency, cone dysfunction, cone-rod dystrophy, and BED were prioritized. Interpretation at the *OPN1LW*/*OPN1MW* locus considered paralog assignment, exon location, inheritance, array configuration, and the clinical phenotype.

Variant interpretation followed ACMG/AMP guidelines, incorporating population frequency, predicted consequence, conservation, segregation, inheritance, phenotype, and published evidence [67]. Nomenclature followed HGVS recommendations [68], using *OPN1LW* NM_020061.6 and *OPN1MW* NM_000513.2. Missense pathogenicity and conservation were assessed using VarCards [69], including CADD, PolyPhen-2, SIFT, MutationTaster, GERP++, phyloP, phastCons, and SiPhy when available.

Candidate sequence variants were validated by Sanger sequencing, with segregation analysis in available relatives. Sanger primers were designed so that each amplicon was *OPN1LW*-specific, either through L-specific primer sites or through an L-specific outer PCR in a nested design. Primer sequences, binding positions, L/M-discriminating nucleotides, PCR strategy and expected product sizes are listed in Supplementary Table S6.

### 4.4. Copy-Number Analysis

MLPA was performed in F7-1 using SALSA MLPA Probemix P497 Opsin (MRC Holland, Amsterdam, The Netherlands) targeting *OPN1LW*, *OPN1MW*, and the LCR. The analysis was performed with sex-matched normal controls and the software-applied interpretation range for X-linked hemizygous targets (0.7–1.3); relative peak ratios outside this range were interpreted as exon dosage alterations. To further assess the *OPN1LW* exon 2–5 deletion in F10-1 and his daughter, quantitative PCR (qPCR) was performed targeting *OPN1LW* exons 2, 4, and 5 with primers containing L/M-discriminating nucleotides (Supplementary Table S6). Two unaffected female controls were included, and the *ALB* gene was used as the reference for normalization. All reactions were performed in triplicate, and relative dosage was calculated by the comparative cycle-threshold method; mean values and standard deviations are shown in Figure 3A. Results were compared with the WES findings.

### 4.5. Nanopore Sequencing and Array Analysis

Oxford Nanopore sequencing was performed in selected unresolved cases with suspected copy-number changes, exon deletions, LCR rearrangements, hybrid genes, or ambiguous array configurations when samples were accessible; five individuals were sequenced (F3-1, F3-1F, F8-1, F9-1 and F9-1F). Samples from F7-1 and F10-1 were not accessible for long-read sequencing. Approximately 1 μg of genomic DNA underwent repair with NEBNext FFPE Repair Mix (M6630; New England Biolabs, Ipswich, MA, USA). This step was included to repair nicks and damaged bases in blood-derived DNA before library preparation. End repair and dA-tailing used the NEBNext Ultra II End Repair/dA-Tailing Module (E7546; New England Biolabs, Ipswich, MA, USA). Adapter ligation used the NEBNext Quick Ligation Module (E6056; New England Biolabs, Ipswich, MA, USA) and the AMX-F adapter supplied with the ONT Ligation Sequencing Kit (SQK-LSK110; Oxford Nanopore Technologies, Oxford, UK). AMPure XP beads (A63882; Beckman Coulter, Brea, CA, USA) were used for purification after end preparation and ligation. Libraries were quantified with the Qubit dsDNA HS Assay Kit (Q32854; Life Technologies Corporation, Eugene, OR, USA), loaded onto FLO-PRO002 flow cells, and sequenced on a PromethION 48 instrument (Oxford Nanopore Technologies, Oxford, UK). No target-capture enrichment was performed. Basecalling used Guppy v6.2.1 with the high-accuracy (HAC) model, and reads passing Q ≥ 7 were retained for analysis. For each sample, read N50 ranged from 16.9 to 20.6 kb, and mean genomic coverage ranged from 22.7 to 40.0-fold.

Reads were aligned independently to GRCh37 (hg19) and GRCh38 (hg38) using the Nanopore preset in minimap2 (https://github.com/lh3/minimap2; accessed on 16 September 2026), generating dual BAM alignments; reported coordinates follow the GRCh38 alignment where applicable, and cross-assembly consistency was verified using the UCSC Genome Browser liftOver tool (https://genome.ucsc.edu/cgi-bin/hgLiftOver; accessed on 16 September 2026). SAMtools (https://www.htslib.org/; accessed on 16 September 2026) was used to convert, sort, and index the alignments. Structural variants were called with Sniffles (https://github.com/fritzsedlazeck/Sniffles; accessed on 16 September 2026), annotated with SnpEff (https://pcingola.github.io/SnpEff/; accessed on 16 September 2026), and reviewed in Integrative Genomics Viewer (https://igv.org/doc/desktop/; accessed on 16 September 2026) [70,71,72,73,74]. Manual review considered breakpoint-spanning and split reads, soft clipping, local depth, LCR involvement, exon dosage and possible hybrid configurations. A minimum of five high-quality ONT reads was required to support a molecular call; F8-1 and F9-1 each had more than ten supporting reads. Findings were interpreted alongside available short-read results, family evidence and phenotype. Sanger validation was used to corroborate findings in locally low-depth regions. When complete spanning reads were unavailable, interpretation was therefore based on reads spanning exon 3 to exon 5 or on breakpoint-spanning reads.

### 4.6. Protein Analysis

The human *OPN1LW* model AF-P04000-F1 was obtained from the AlphaFold Protein Structure Database [75,76]. Missense-variant positions, predicted transmembrane domains and local structural environments were examined in PyMOL V3.1.9 (Schrödinger, LLC, New York, NY, USA; https://pymol.org/; accessed on 16 September 2026). Potential structural consequences were evaluated using HOPE (https://www3.cmbi.umcn.nl/hope/; accessed on 16 September 2026) [77]. Conservation was assessed by multiple sequence alignment in Lasergene v17.2 (DNASTAR, Madison, WI, USA). These analyses provided structural context and were not treated as experimental functional evidence or independently combined pathogenicity criteria. Previously reported missense-variant sites shown in Figure 3C were obtained from HGMD [48].

Sequence-based physicochemical properties calculated with ExPASy ProtParam (https://web.expasy.org/protparam/; accessed on 16 September 2026) are listed for descriptive purposes only (Supplementary Table S7) [44,45,46,77,78,79,80]; they are not measurements of protein stability and were not used as evidence.

## Figures and Tables

**Table 1:** Clinical findings in 12 patients with *OPN1LW*/*OPN1MW*-associated disorders.

Clinical Item		F1-1	F2-1	F2-2	F2-3	F3-1	F4-1	F5-1	F6-1	F7-1	F8-1	F9-1	F10-1
Age at first visit/sex		1.00/M	5.00/M	8.00/M	59.00/M	8.00/M	4.92/M	7.00/M	1.00/M	8.00/M	3.00/M	4.00/M	78.00/M
Clinical diagnosis		BCM	BCM	BCM	BCM	BCM	XLCOD	XLCOD	XLCOD, eoHM	XLCOD	BED	BED	CVD
Visual acuity	OD	0.5	0.3	0.3	0.01	0.15	0.2	0.3	0.3	0.6	0.9	0.5	0.4
	OS	0.5	0.4	0.3	0.05	0.15	0.2	0.2	0.3	0.6	0.7	0.6	0.4
	Age	(5.89 y)	(7.10 y)	(8.81 y)	(59.43 y)	(8.57 y)	(4.92 y)	(7.16 y)	(6.24 y)	(8.63 y)	(8.42 y)	(7.34 y)	(78.85 y)
Refraction	OD	−7.50/−3.50 × 10	−6.50/−2.25 × 180	−2.75/−3.25 × 175	−11.00 DS	−5.75/−1.75 × 10	−3.25/−2.50 × 10	+2.50/−2.00 × 15	−13.50/−3.00 × 180	+1.50/−0.75 × 20	−7.25/−1.00 × 7	−8.50/−1.75 × 20	+1.00/−0.50 × 65
	OS	−8.50/−3.50 × 180	−7.25/−2.25 × 175	−3.25/−2.25 × 180	−10.00 DS	−6.50/−1.50 × 10	−2.75/−2.50 × 170	+2.00/−1.50 × 170	−13.00/−3.00 × 165	+1.50/−0.50 × 170	−7.00/−2.50 × 160	−8.50/−1.50 × 175	+1.50/−1.00 × 70
	Age	(5.89 y)	(7.10 y)	(8.81 y)	(59.43 y)	(8.57 y)	(4.92 y)	(6.13 y)	(2.98 y)	(8.62 y)	(8.42 y)	(7.34 y)	(78.85 y)
AL, mm		NR	OD: 26.20 OS: 26.45 (4.87 y)	OD: 24.70 OS: 24.79 (6.00 y) OD: 25.20 OS: 25.28 (8.00 y)	NR	NR	OD: 26.25 OS: 25.96 (4.92 y)	OD: 24.20 OS: 24.15 (7.16 y)	OD: 25.56; OS: 25.15 (1.80 y) OD: 26.89; OS: 26.71 (2.97 y) OD: 28.55; OS: 28.38 (6.24 y)	OD: 23.68 OS: 24.59 (7.54 y)	OD: 27.56 OS: 27.78 (8.36 y)	OD: 27.47; OS: 27.30 (7.34 y) OD: 27.70; OS: 27.36 (10.00 y)	NR
Color vision/D-15		(1) Plates: partly failed (2) D-15: fail, P/D axis (2.82 y)	(1) Plates: blue digits only (2) D-15: fail, P/D axis (4.87 y)	D-15: fail, P/D axis (8.81 y)	D-15: fail, mixed/indeterminate (59.43 y)	D-15: fail, P/D axis (8.57 y)	D-15: fail, P/D axis (4.92 y)	(1) Most plates unrecognized (2) D-15: fail, P/D axis (7.16 y)	(1) Plates: partly failed (2) D-15: borderline/near-normal (3) No definite axis (3.62 y)	(1) Abnormal blue recognition (2) D-15: fail, mixed/indeterminate (8.63 y)	(1) Most plates failed (2) Marked red-green defect (3) D-15: NR (6.40 y)	Red-green defect (5.31 y)	(1) Plates: partial failure (2) Red-green defect (3) D-15: NR (78.85 y)
Fundus		(1) Tessellated fundus (2) Mildly tilted discs (3) PPA (4.76 y)	(1) Tilted discs (2) PPA (3) Tessellated fundus (4.87 y)	(1) Pale discs (8.81 y)	(1) Tilted optic discs /PPA (2) macular atrophy (3) Peripheral pigmentation (59.43 y)	(1) Tessellated fundus (2) Tilted disc (3) Mild mid-peripheral mottling (8.57 y)	(1) Tessellated fundus (2) PPA (4.86 y)	(1) Tilted discs (2) Poor foveal reflex (7.16 y)	(1) Tessellated fundus (2) Tilted discs (2.97 y)	Poor foveal reflex (8.63 y)	(1) Tilted discs (2) PPA (3) Tessellated fundus (8.42 y)	(1) Tilted discs (2) PPA (3) Tessellated fundus (6.21 y)	(1) Tessellated fundus (2) Temporal disc pallor (78.85 y)
OCT		Unremarkable (5.89 y)	Foveal EZ/IZ blurring (7.10 y)	Focal EZ/IZ disruption (8.81 y)	(1) Macular atrophy (2) Diffuse retinal thinning with disrupted outer retinal layers. (59.43 y)	EZ disruption (8.57 y)	EZ disruption (4.86 y)	EZ blurring (7.16 y)	Foveal IZ disruption (3.62 y)	Normal (8.63 y)	Foveal IZ disruption (8.42 y)	(1) Posterior staphyloma (2) Foveal EZ/IZ blurring (8.35 y)	Foveal IZ disruption (78.85 y)
FAF		Unremarkable (4.76 y)	NR	Unremarkable (8.81 y)	Matching hypo-AF (59.43 y)	Mild irregular Hypo-AF in the macular region and mid-periphery. (8.57 y)	NR	Unremarkable (7.16 y)	Hyper-AF foveal reflex (4.57 y)	Normal (8.63 y)	hyper-AF foveal reflex (8.42 y)	Extensive posterior pole hypo-AF zone (7.34 y)	NR
ffERG		(1) Cone: near-extinguished/severely decreased (2) Rod: moderately decreased (5.89 y)	NR	NR	(1) Rod: severely decreased (2) Photopic 3.0/30 Hz: extinguished (59.53 y)	(1) DA waveforms: normal (2) Photopic 3.0/30-Hz: near-extinguished (8.57 y)	Photopic/flicker: markedly decreased (near-extinguished) (4.92 y)	(1) DA: largely preserved (2) OPs: moderately decreased (3) Photopic/30 Hz: moderately decreased (7.19 y)	Markedly reduced cone responses (4.14 y)	Cone responses decreased (8.64 y)	(1) DA: normal (2) Photopic 3.0/30 Hz: mild-moderately decreased (8.87 y)	(1) DA: normal (2) OPs: moderately decreased (3) Photopic/30 Hz: moderately decreased (6.21 y)	NR
S-cone ERG		NR	NR	NR	Amplitudes decreased (59.53 y)	NR	NR	NR	NR	Recorded; descriptive only. (8.64 y)	NR	NR	NR
Other ocular phenotype		-	-	(1) Strabismus (2) Surgery at 5.00 y	(1) Cataract (2) Retinal hole	Vitreous opacities	-	-	-	-	-		Cataract
Family history		(1) Maternal uncles/cousins: CVD (2) Some had poor VA	Maternal grandfather: HM, poor VA	Maternal grandfather: poor VA	Two similarly affected grandsons; brother/uncle with poor VA.	NR	NR	Maternal grandfather reported as affected; the familial variant was identified in the proband, mother and maternal grandfather; the older brother tested negative.	Multiple relatives: myopia/HM	(1) Maternal uncle: CVD (2) Parents: normal VA	(1) Mother/cousin: myopia/HM (2) Maternal great-grandfather: poor VA	Maternal uncle: HM+ CVD	Three grandsons: similar CVD

Note: Age at first visit/sex denotes age at first visit. Examination ages are given in decimal years in parentheses after each ophthalmic assessment result, e.g., (5.89 y). Unreliable F1-1 biometry was excluded and is shown as NR. NR means not recorded and does not imply a normal or negative result. BCM, blue cone monochromacy; XLCOD, X-linked cone dystrophy; BED, Bornholm eye disease; CVD, color vision deficiency; eoHM, early-onset high myopia; HM, high myopia; DA, dark adapted; ERG, electroretinography; OPs, oscillatory potentials; OCT, optical coherence tomography; FAF/AF, fundus autofluorescence/autofluorescence; EZ/IZ, ellipsoid zone/interdigitation zone; PPA, peripapillary atrophy; P/D, protan/deutan; VA, visual acuity; AL, axial length; OD/OS, right/left eye.

## Data Availability

The original contributions presented in this study are included in the article/Supplementary Material. Further inquiries can be directed to the corresponding author.

## Supplementary Materials

The following supporting information can be downloaded at: https://www.mdpi.com/article/10.3390/ijms27219705/s1.
