# Supplementary material for: Clinical and Genetic Spectrum of OPN1LW/OPN1MW-Associated Disorders in a Chinese Cohort: Contribution of Long-Read Sequencing
Source: Int J Mol Sci. 2026 Oct 29;27(21):9705. doi: 10.3390/ijms27219705 (PMC13763260; doi:10.3390/ijms27219705)
Supplement: Supplementary file 1 [file ijms-27-09705-s001.zip › ijms-4608590-supplementary.pdf]

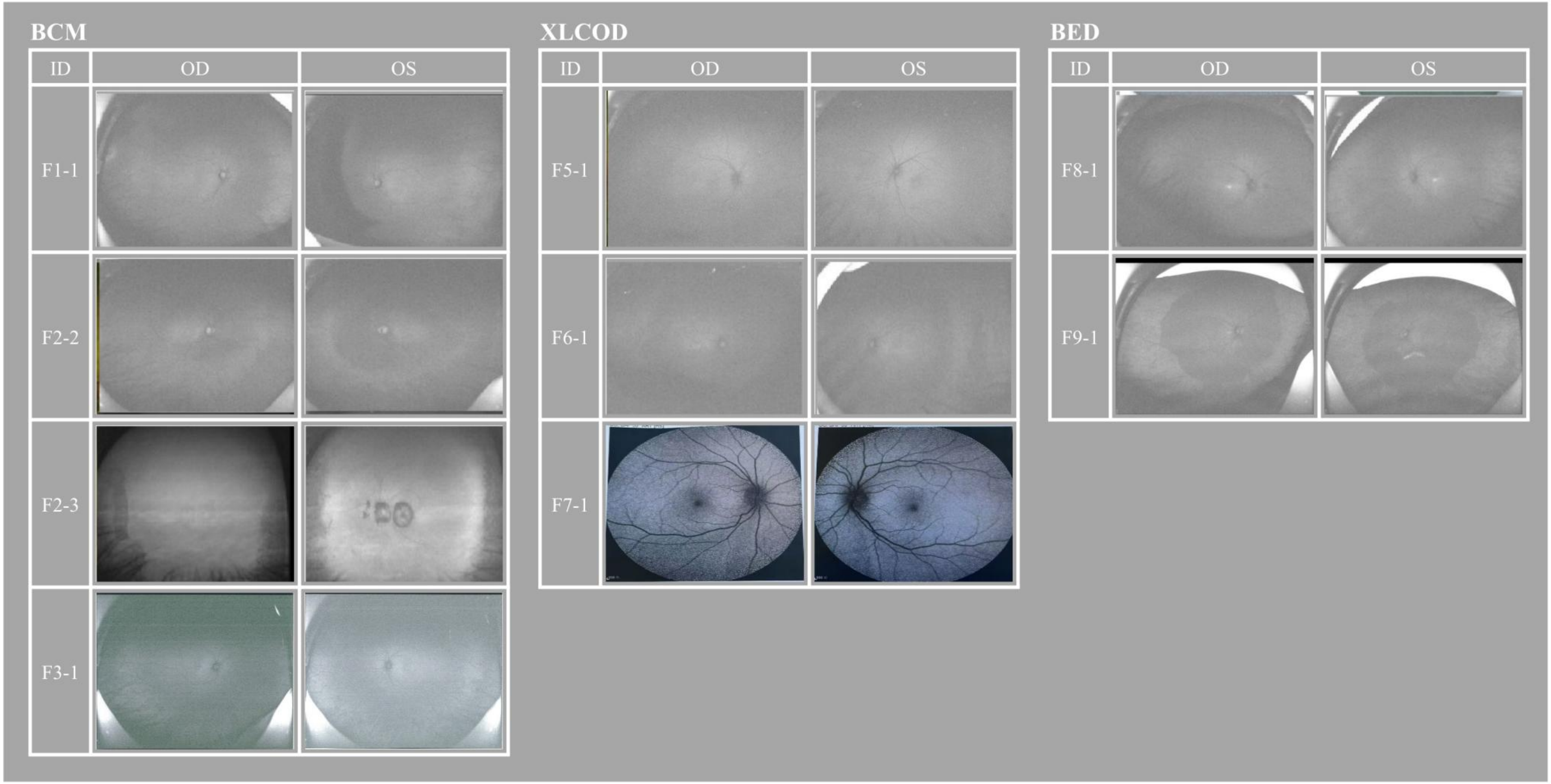

Supplementary Figure S1. Fundus autofluorescence in *OPN1LW/OPN1MW*-associated patients. Short wavelength fundus autofluorescence images of the right (OD) and left (OS) eyes are shown for the nine patients in whom autofluorescence imaging was available; patients are grouped by clinical diagnosis and ordered by identifier. Findings ranged from an unremarkable pattern to a hyperautofluorescent foveal reflex, matching hypoautofluorescence, and an extensive posterior-pole hypoautofluorescent zone. The corresponding fundus and OCT images are shown in Figure 1, and the full clinical dataset in Table 1. FAF images for F7-1 were acquired with a Heidelberg Engineering system; the remaining FAF images used an Optos system.

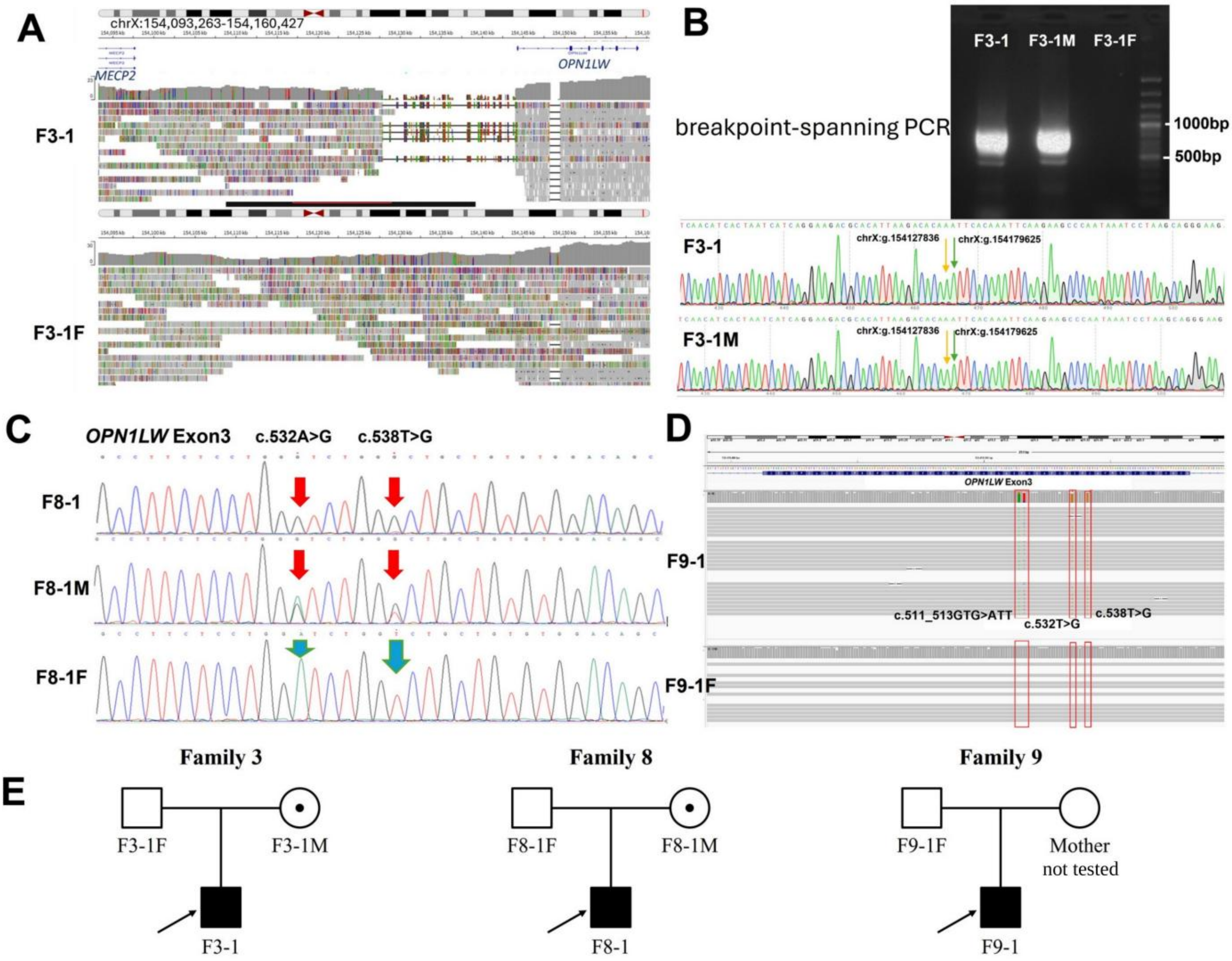

Supplementary Figure S2. Pedigree plots and co-segregation analysis in three families investigated by Oxford Nanopore long-read sequencing. (A) Long-read alignments for F3-1 and his father (F3-1F) across the deletion region. (B) Breakpoint-spanning PCR and Sanger sequencing of the deletion junction in Family 3. (C) Sanger sequencing of OPN1LW exon 3 in F8-1 and his parents. (D) Long-read alignments of *OPN1LW* exon 3 in F9-1 and his father (F9-1F). (E) Pedigrees of Families 3, 8 and 9. Squares denote males, circles females, filled symbols affected individuals, and central dots confirmed heterozygous carriers. Arrows identify the probands.

Supplementary Table S1. Serial axial-length measurements in F6-1

Examination date	Age (years)	OD (mm)	OS (mm)
19 Oct 2020	1.80	25.56	25.15
20 Dec 2021	2.97	26.89	26.71
16 Aug 2022	3.62	27.26	27.09
20 Feb 2023	4.13	27.58	27.41
31 Jul 2023	4.57	27.78	27.62
31 Mar 2025	6.24	28.55	28.38

Values at 2.97, 3.62, 4.13 and 4.57 years were taken from IOLMaster 700 (Carl Zeiss Meditec AG) reports; values at 1.80 and 6.24 years were abstracted from clinical records, in which the device was not documented. Figure 2C plots the same values.

Supplementary Table S2. Patient-level molecular assays, findings, genomic coordinates and interpretation limits

Patient	Assays reported in the records	Molecular finding	Basis of interpretation and remaining limits
F1-1	WES; Sanger	*OPN1LW* c.739C>T p.(Arg247Ter); Likely pathogenic[1] chrX:g.154154734C>T (GRCh38) chrX:g.153420209C>T (GRCh37)	Sequence variant confirmed by *OPN1LW*-specific Sanger sequencing; array configuration (*OPN1MW* copy number) not fully resolved. The trace containing the variant also shows L-type bases at exon 4 codons 230, 233 and 236.
F2-1	WES; Sanger	*OPN1LW* c.161dup p.(His54GlnfsTer46); Pathogenic[1] chrX:g.154150704dup (GRCh38) chrX:g.153416176dup (GRCh37)	Sequence variant confirmed by *OPN1LW*-specific Sanger sequencing; array configuration (*OPN1MW* copy number) not fully resolved.
F2-2	WES; Sanger	*OPN1LW* c.161dup p.(His54GlnfsTer46); Pathogenic[1] chrX:g.154150704dup (GRCh38) chrX:g.153416176dup (GRCh37)	Sequence variant confirmed by *OPN1LW*-specific Sanger sequencing; array configuration (*OPN1MW* copy number) not fully resolved.
F2-3	WES; Sanger	*OPN1LW* c.161dup p.(His54GlnfsTer46); Pathogenic[1] chrX:g.154150704dup (GRCh38) chrX:g.153416176dup (GRCh37)	Sequence variant confirmed by *OPN1LW*-specific Sanger sequencing; array configuration (*OPN1MW* copy number) not fully resolved.
F3-1	WES; WGS; ONT; junction PCR/Sanger	LCR-containing deletion chrX:g.154127837_154179624del (hg38; 51,788 bp) chrX:g.153393306_153445112del (hg19; 51,807 bp)	Long-read junction and PCR/Sanger evidence reported; product in patient and mother, not father. The deletion removes the LCR and *OPN1LW*. Not formally classified under the ACMG/ClinGen CNV framework.
F4-1	WES; Sanger	*OPN1LW* c.151T>C p.(Trp51Arg); VUS[1] chrX:g.154150694T>C (GRCh38) chrX:g.153416166T>C (GRCh37)	Sequence variant confirmed by *OPN1LW*-specific Sanger sequencing; array configuration (*OPN1MW* copy number) not fully resolved. In silico predictions do not establish functional effect.
F5-1	WES; Sanger	*OPN1LW* c.559C>T p.(Pro187Ser); Likely pathogenic[1] chrX:g.154153089C>T (GRCh38) chrX:g.153418562C>T (GRCh37)	Sequence variant confirmed by *OPN1LW*-specific Sanger sequencing; array configuration (*OPN1MW* copy number) not fully resolved. External functional evidence is distinct from patient-specific validation.
F6-1	WES; Sanger	*OPN1LW* c.1A>C p.(Met1?); Likely pathogenic[1] chrX:g.154144284A>C (GRCh38) chrX:g.153409758A>C (GRCh37)	Sequence variant confirmed by *OPN1LW*-specific Sanger sequencing; array configuration (*OPN1MW* copy number) not fully resolved.
F7-1	WES; MLPA	*OPN1LW* exon 3–5 dosage decrease; increased *OPN1MW* dosage	Dosage patterns suggest a structural rearrangement, possibly a hybrid gene, but do not resolve hybrid sequence identity or complete array configuration. Not formally classified under the ACMG/ClinGen CNV framework.
F8-1	WES; WGS; ONT; Sanger	*OPN1LW*-LVAVA / *OPN1MW*-MVAVA Exon 3 haplotypes	Long-read sequencing resolved the proband's haplotypes; the maternal *OPN1LW* allele was confirmed by Sanger, whereas the maternal *OPN1MW* haplotype was inferred from WES and not fully confirmed.

Patient	Assays reported in the records	Molecular finding	Basis of interpretation and remaining limits
F9-1	WES; WGS; ONT	*OPN1LW*-LIAVA / *OPN1MW*-MVVVA<br>Exon 3 haplotypes	Long-read sequencing resolved haplotypes that short-read sequencing could not reliably dissect. Segregation was not assessed: the mother was not tested, and the father's haplotypes do not inform X-linked transmission to a son.
F10-1	WES; qPCR	*OPN1LW* exon 2–5 deletion (inferred)	The *OPN1LW* exon 2–5 deletion inferred from WES data was assessed by qPCR at exons 2, 4 and 5: no L-specific signal was detected in F10-1, and his daughter showed about half of the control dosage. A hybrid gene cannot be excluded. Not formally classified under the ACMG/ClinGen CNV framework.

WES, whole-exome sequencing; WGS, whole-genome sequencing; ONT, Oxford Nanopore sequencing; MLPA, multiplex ligation-dependent probe amplification; qPCR, quantitative PCR; LCR, locus control region. Reference transcripts: *OPN1LW* NM_020061.6; *OPN1MW* NM_000513.2. Genomic coordinates are reference mappings of NM_020061.6 on GRCh38 (NC_000023.11) and GRCh37 (NC_000023.10).

Supplementary Table S3. Computational predictions and evolutionary conservation annotations for OPN1LW c.151T>C, p.(Trp51Arg), identified in F4-1

Algorithm or annotation	Score	Interpretation	Threshold or interpretation rule	Ref.
SIFT	0	Deleterious	Score ≤0.05 in commonly used dbNSFP annotations; lower scores indicate less tolerance.	[27–28]
PolyPhen-2 HumDiv	0.975	Probably damaging	Benign: 0–0.452; possibly damaging: 0.453–0.956; probably damaging: 0.957–1.	[19,28]
PolyPhen-2 HumVar	0.835	Possibly damaging	Benign: 0–0.446; possibly damaging: 0.447–0.908; probably damaging: 0.909–1.	[19,28]
LRT	0	Deleterious (D)	Deleterious category: P <0.001, ω <1, ≥10 aligned eutherian species, and the alternative amino acid absent from the alignment. The score is shown rounded (LRT P <0.001; ω = 0); dbNSFP LRT_pred = D.	[29–30]
MutationTaster	1	Disease causing (D; simple_aae model)	The probability and the A/D/N/P category must be interpreted together. A native probability of 1 is not a 100% clinical pathogenicity probability.	[30–31]
MutationAssessor	3.215	Medium functional impact	Classic FI boundaries are approximately 0.8, 1.9 and 3.5; some annotation releases use 1.935 for the middle boundary. This score is medium under either convention.	[32–33]
FATHMM	1.22	Tolerated	For the classic weighted missense model, the decision boundary is −1.5; lower scores favor a damaging prediction.	[20]
PROVEAN	−6.01	Deleterious	Score ≤−2.5: deleterious; score >−2.5: neutral.	[34]
VEST3	0.884	Continuous score; higher values favor functional impact	Not dichotomized. No universal clinical classification threshold is assigned to this retained score.	[33,35]
MetaSVM	−0.63	Tolerated	The native score boundary is 0; higher scores favor a damaging prediction.	[21,33]
MetaLR	0.204	Tolerated	The native logistic-regression score boundary is 0.5.	[21,33]
M-CAP	0.175	Above the high-sensitivity screening threshold	The original study used >0.025 as a screening threshold. Exceeding this value does not establish clinical pathogenicity.	[36]
CADD	23.5	PHRED-scaled; within the top 1% of scored reference-genome SNVs	PHRED ≥20 denotes the highest-scoring 1% of possible reference-genome SNVs. The value is the PHRED-scaled score (raw C-score 3.89).	[37]
DANN	0.991	Continuous score; higher values favor predicted deleteriousness	Range 0–1; not dichotomized in this table.	[38]
FATHMM-MKL	0.941	Predicted deleterious	The coding-model decision boundary is 0.5; higher scores favor deleteriousness. This is a different scale from classic FATHMM.	[39]
GenoCanyon	1	High functional potential of the genomic position	Position-level functional annotation; not a variant-specific pathogenicity classification.	[40]
GERP	4.11	Positive evolutionary constraint score	GERP++ rejected-substitution (RS) score; reported continuously without a clinical cutoff.	[41]
phyloP	1.935	Positive conservation score	phyloP100way_vertebrate score; positive values indicate conservation and negative values indicate acceleration.	[42]
phastCons	0.981	High posterior probability of membership in a conserved element	phastCons100way_vertebrate; basewise scores range from 0 to 1. This is a conservation annotation, not a pathogenicity probability.	[43]
SiPhy	11.575	Continuous evolutionary constraint score	SiPhy 29-way (mammalian) log-odds score; not dichotomized.	[44]
REVEL	0.417	Indeterminate for PP3/BP4 under the cited calibration	Pejaver et al. (2022): 0.290 < score < 0.644 is the indeterminate interval. This score supplies neither PP3 nor BP4 under that scheme.	[22,45]
ReVe	0.996	High relative rank; above the original screening threshold	The original study used >0.86 to screen deleterious missense variants. ReVe integrates ranked REVEL and VEST3 outputs and is not independent of them.	[46]

Reference numbers refer to the reference list of the main article.

Supplementary Table S4. Sequencing quality metrics of ONT long-read and short-read data

(a) ONT long-read sequencing

Sample ID	Sex	Total bases (Gb)	Total reads (M)	Mean depth (×, whole genome)	Genome coverage (%)	Mean quality (Q/Phred)	Bases ≥Q30 (%)	Est. coverage (×)	Read N50 (bp, whole genome)	Mean depth, chrX (×)	Mean read length at Opsin locus (bp)	ONT reads spanning exon 3 to exon 5 * (*OPN1LW* identity; *OPN1MW* identity)
F8-1	XY	99.77	4.99	30.44	92.35%	21.00	15.14%	32.17	17,614	17.56	17304.00	13; 5
F9-1	XY	102.92	4.68	31.19	92.34%	21.00	15.63%	33.18	20,629	18.07	21375.00	18; 3
F9-1F	XY	131.99	6.61	40.01	92.34%	20.70	15.13%	42.55	16,853	23.22	26276.00	9; 15
F3-1	XY	74.67	3.19	22.72	92.31%	20.00	12.49%	24.07	20,347	13.12	30488.00	0; 4
F3-1F	XY	103.39	5.14	31.31	92.24%	21.50	13.98%	33.33	17,491	18.52	23842.00	11; 2

* Reads were counted in the GRCh38 alignment using primary alignments only. Reads aligned uniquely to *OPN1LW* or *OPN1MW* were counted separately and are shown as *OPN1LW* identity; *OPN1MW* identity. Multi-mapped reads that could not distinguish the two genes were excluded. No *OPN1LW*-identity reads are listed for F3-1 because *OPN1LW* is entirely deleted in this patient. The five ONT-sequenced individuals are F3-1, F3-1F, F8-1, F9-1 and F9-1F.

(b) Short-read WES and WGS

Sample ID	Platform	Sex	Mean depth (×)	Mean depth, chrX (×)	% target regions ≥20×
F8-1	WGS	XY	50.11	24.41	99.58%
F8-1M	WGS	XX	52.68	49.33	98.90%
F8-1F	WGS	XY	52.83	25.68	99.58%
F9-1	WGS	XY	49.78	25.42	99.49%
F9-1M	WGS	XX	52.29	48.82	98.90%
F9-1F	WGS	XY	45.76	22.18	99.39%
F8-1	WES	XY	161.98	88.23	99.94%
F8-1M	WES	XX	157.10	160.04	99.75%
F8-1F	WES	XY	169.32	109.52	99.92%
F9-1	WES	XY	137.90	75.15	99.94%
F9-1M	WES	XX	164.31	198.91	99.74%
F9-1F	WES	XY	149.84	97.25	99.90%

Supplementary Table S5. Uniquely-mapped coverage of *OPN1LW*/*OPN1MW* exons in short-read sequencing data

Region / exon	Mean depth (×), all samples (mean±SD)	Median depth (×)	% bases ≥20× (mean±SD)	% bases with 0× (mean±SD)	WGS mean depth (×) (mean±SD)	WES mean depth (×) (mean±SD)
OPN1LW locus	13.4±8.2	11.8	16.67%	62.66%	12.1±5.2	14.7±10.9
OPN1LW exon 1	52.6±52.3	41	57.08%	0.00%	15.8±4.6	89.5±52.3
OPN1LW exon 2	117.2±134.4	60.8	92.51%	0.00%	31.9±13.3	202.5±148.7
OPN1LW exon 3	1.3±4.4	0	0.00%	91.67%	2.5±6.2	0.0±0.0
OPN1LW exon 4	90.1±104.1	50.2	88.00%	0.00%	27.4±11.5	152.8±119.5
OPN1LW exon 5	138.5±166.6	68.2	95.63%	0.00%	30.1±10.4	246.8±181.2
OPN1LW exon 6	1.1±3.9	0	0.69%	91.67%	2.2±5.5	0.0±0.0
OPN1MW locus	6.6±4.2	4.8	7.68%	72.11%	7.1±4.2	6.2±4.5
OPN1MW exon 1	1.1±3.8	0	0.00%	91.67%	2.2±5.4	0.0±0.0
OPN1MW exon 2	31.8±49.7	14.2	26.79%	10.27%	8.6±5.4	55.0±64.1
OPN1MW exon 3	1.2±4.1	0	0.00%	91.67%	2.4±5.8	0.0±0.0
OPN1MW exon 4	1.0±2.7	0	0.00%	76.56%	2.0±3.7	0.1±0.2
OPN1MW exon 5	117.2±150.1	44.4	90.14%	8.33%	34.8±9.7	199.7±182.0
OPN1MW exon 6	1.2±4.1	0	0.07%	91.67%	2.4±5.8	0.0±0.0
MECP2 (control)	31.7±12.7	24.4	81.36%	0.03%	31.7±12.7	—
DMD (control)	35.6±14.5	27.6	89.84%	0.04%	35.6±14.5	—

Note. Uniquely-mapped coverage (MAPQ≥20) derived from the DP field of gVCFs generated by GATK HaplotypeCaller (default minMappingQualityScore=20). Data are calculated from families for whom both short-read NGS and ONT long-read sequencing data are available.

Supplementary Table S6. Primers used for Sanger sequencing and qPCR, with binding positions and L/M-discriminating nucleotides

Target variant or junction	Primer name	Primer sequence 5′ to 3′	Product size, bp	PCR strategy	Binding interval and strand (chrX, GRCh37)[2]	Specificity and L/M-discriminating nucleotides[2]
c.739C>T	*OPN1LW*_E2-E4 FL	TCAGGACAGCCGTCATAT TGGA	7790	First-round nested PCR	153414081–153414102 (+)	*OPN1LW* intron 1; no *OPN1MW* site with <6 mismatches; outer pair is L-specific.
c.739C>T	*OPN1LW*_E2-E4 RL	TCCCCAGCAGACGCAGTA CGCAAAGAT	7790	First-round nested PCR	153421844–153421870 (−)	*OPN1LW* exon 5; *OPN1MW* differs at bases 12, 17, 19, 22, 24 and 27 (C/A, T/A, C/T, A/C, A/G, T/C).
c.739C>T	*OPN1LW*_E4F1	AGCACGATCAGGTCACAG GC	1819	Second-round nested PCR	153418757–153418776 (+)	Identical in *OPN1MW*; specificity conferred by the L-specific outer PCR.
c.739C>T	*OPN1LW*_E4R1	TGGCTGCTCCACTCTACT CTGT	1819	Second-round nested PCR	153420554–153420575 (−)	Identical in *OPN1MW*; specificity conferred by the L-specific outer PCR. The sequenced amplicon also shows L-type codons 230, 233 and 236.
c.161dup	*OPN1LW*_E2F	CAGCTACTACTCCGGAGG CT	1690	Single-round PCR	GRCh38: 154149413–154149432 (+)[3]	*OPN1LW* intron 1; no *OPN1MW* site with <6 mismatches; confers L specificity to the E2F/E2R pair.
c.161dup	*OPN1LW*_E2R	CCAGAACCCTTCAGTATA TGGATG	1690	Single-round PCR	153416551–153416574 (−)	Identical in *OPN1MW*; specificity conferred by *OPN1LW*_E2F.
c.151T>C	*OPN1LW*_E2F	CAGCTACTACTCCGGAGG CT	1690	Single-round PCR	GRCh38: 154149413–154149432 (+)[3]	*OPN1LW* intron 1; no *OPN1MW* site with <6 mismatches; confers L specificity to the E2F/E2R pair.
c.151T>C	*OPN1LW*_E2R	CCAGAACCCTTCAGTATA TGGATG	1690	Single-round PCR	153416551–153416574 (−)	Identical in *OPN1MW*; specificity conferred by *OPN1LW*_E2F.
c.559C>T	*OPN1LW*_E3F	CCAGTGTCTGTTCGAGGT TGGT	1943	Single-round PCR	153418244–153418265 (+)	Identical in *OPN1MW*; specificity conferred by *OPN1LW*_E3R.
c.559C>T	*OPN1LW*_E3R	GGTAGCAGAGCATGATGA GAGC	1943	Single-round PCR	153420167–153420188 (−)	*OPN1LW* exon 4 (c.697–718); one mismatch to both genes at base 19; *OPN1MW* differs at bases 13, 20, 21 and 22 (T/C, A/G, G/C, C/T; codons 233 and 236).
c.1A>C	*OPN1LW*_E1F	AATCCCTCTGAGCCGCCC TTGC	2252	Single-round PCR	153409533–153409554 (+)	*OPN1LW* 5′ flank; *OPN1MW* differs at bases 19, 20 and 22 (T/C, T/C, C/G).

Target variant or junction	Primer name	Primer sequence 5′ to 3′	Product size, bp	PCR strategy	Binding interval and strand (chrX, GRCh37)[2]	Specificity and L/M-discriminating nucleotides[2]
c.1A>C	*OPN1LW*_E1R	CAAAACACACCAACAAATCCAACCTA	2252	Single-round PCR	153411759–153411784 (−)	*OPN1LW* intron 1; *OPN1MW* differs at the 3′-terminal base 26 (A/G).
F3-1 deletion junction[1]	chrX-del-F1	TAAGCCATGTGTGTACCACTG	999	Breakpoint-junction PCR validation	153392808–153392828 (+)	Deletion-junction forward primer; specific to the region upstream of *OPN1LW*.
F3-1 deletion junction[1]	chrX-del-R1	TTGATGCTATTGAGAAGGCAG	999	Breakpoint-junction PCR validation	153445564–153445584 (−)	Matched upstream of *OPN1MW*/MW2/MW3; the breakpoint-spanning pair is specific.
OPN1LW exon 2 dosage (F10-1)	*OPN1LW*_E2F1-QPCR	ATCAGCATTGTGAACCAGGTCTC	160	qPCR	153416340–153416362 (+)	*OPN1MW* differs at bases 7 and 23 (A/G, C/A).
OPN1LW exon 2 dosage (F10-1)	*OPN1LW*_E2R1-QPCR	TCATCATGAGGTGGCTGGAGGA	160	qPCR	153416478–153416499 (−)	*OPN1MW* differs at bases 16 and 22 (T/C, A/G).
OPN1LW exon 4 dosage (F10-1)	*OPN1LW*_E4F2-QPCR	GCTGCATCATCCCACTCGCTA	94	qPCR	153420150–153420170 (+)	*OPN1MW* differs at bases 10, 18, 19 and 20 (T/C, G/A, C/G, T/C).
OPN1LW exon 4 dosage (F10-1)	*OPN1LW*_E4R2-QPCR	GTGAGGCCAGGAGGAATCGG	94	qPCR	153420224–153420243 (−)	Identical in *OPN1MW*; specificity conferred by *OPN1LW*_E4F2-QPCR.
OPN1LW exon 5 dosage (F10-1)	*OPN1LW*_E5F-QPCR	GGTGGTGGTGATGaTCtTtGCgTa	104	qPCR	153421831–153421854 (+)	*OPN1MW* differs at bases 14, 17, 19, 22 and 24 (A/G, T/C, T/G, G/A, A/T).
OPN1LW exon 5 dosage (F10-1)	*OPN1LW*_E5R-QPCR	CCATCAAAGGGTGGAAGGcGTAa	104	qPCR	153421912–153421934 (−)	*OPN1MW* differs at bases 19 and 23 (C/G, A/G).

[1] F3-1 target description: chrX:g.154127837_154179624del (hg38). The 999-bp product corresponds to the expected amplicon size spanning the predicted deletion region. [2] Binding positions are 1-based and inclusive (GRCh37/hg19 unless stated). L/M differences are given in primer orientation as primer base/*OPN1MW* base, counted from the primer 5′ end. [3] The GRCh37 primary sequence at the corresponding site (chrX:153414886−153414905) differs from the primer at its three 3′-terminal bases. Lower-case letters in qPCR primers are reproduced from the oligonucleotide record. Transcript: *OPN1LW* NM_020061.6.

Supplementary Table S7. Sequence-based physicochemical properties

Protein sequence	Length (aa)	Molecular weight (Da)	Theoretical pI	Instability index
Reference *OPN1LW*	364	40,574.37	8.89	35.10
OPN1LW p.(Trp51Arg) model	364	40,544.35	8.98	35.33

ExPASy ProtParam calculations were recorded on 12 October 2026 for *OPN1LW* UniProtKB P04000 sequence version 2 (364 amino acids; NP_064445.2; NM_020061.6). The variant model differs only at Trp51Arg. The instability indices differ by 0.23; these descriptive estimates do not indicate altered protein stability and were not used as evidence.
